# Body mass index and waist circumference in early adulthood are associated with thoracolumbar spine shape at age 60-64: The Medical Research Council National Survey of Health and Development

**DOI:** 10.1371/journal.pone.0197570

**Published:** 2018-06-14

**Authors:** Anastasia V. Pavlova, Stella G. Muthuri, Rachel Cooper, Fiona R. Saunders, Jennifer S. Gregory, Rebecca J. Barr, Kathryn R. Martin, Judith E. Adams, Diana Kuh, Rebecca J. Hardy, Richard M. Aspden

**Affiliations:** 1 Arthritis and Musculoskeletal Medicine, School of Medicine, Medical Sciences and Nutrition, University of Aberdeen, Aberdeen, United Kingdom; 2 MRC Unit for Lifelong Health and Ageing at UCL, London, United Kingdom; 3 Medicines Monitoring Unit (MEMO), Division of Molecular & Clinical Medicine, School of Medicine, University of Dundee, Dundee, United Kingdom; 4 Manchester Academic Health Science Centre, Central Manchester University Hospitals NHS Foundation Trust, Manchester Royal Infirmary, Manchester, United Kingdom; Medical University of Vienna, AUSTRIA

## Abstract

This study investigated associations between measures of adiposity from age 36 and spine shape at 60–64 years. Thoracolumbar spine shape was characterised using statistical shape modelling on lateral dual-energy x-ray absorptiometry images of the spine from 1529 participants of the MRC National Survey of Health and Development, acquired at age 60–64. Associations of spine shape modes with: 1) contemporaneous measures of total and central adiposity (body mass index (BMI), waist circumference (WC)) and body composition (android:gynoid fat mass ratio and lean and fat mass indices, calculated as whole body (excluding the head) lean or fat mass (kg) divided by height^2^ (m)^2^); 2) changes in total and central adiposity between age 36 and 60–64 and 3) age at onset of overweight, were tested using linear regression models. Four modes described 79% of the total variance in spine shape. In men, greater lean mass index was associated with a larger lordosis whereas greater fat mass index was associated with straighter spines. Greater current BMI was associated with a more uneven curvature in men and with larger anterior-posterior (a-p) vertebral diameters in both sexes. Greater WC and fat mass index were also associated with a-p diameter in both sexes. There was no clear evidence that gains in BMI and WC during earlier stages of adulthood were associated with spine shape but younger onset of overweight was associated with a more uneven spine and greater a-p diameter. In conclusion, sagittal spine shapes had different associations with total and central adiposity; earlier onset of overweight and prior measures of WC were particularly important.

## Introduction

Spinal column and vertebral shape vary greatly between individuals [1–3] and change throughout life. Spinal curvatures help stabilise the body’s centre of mass and develop from childhood to young adulthood, with increases in lumbar lordosis and thoracic kyphosis [4, 5]. However, not only is the timing of development still unclear [6], but we also know little about changes in adulthood, the mechanisms of these changes and what affects them. It is likely that adult spinal shape is influenced by a combination of genetic, environmental and biomechanical factors, but it is difficult to distinguish between these, and further research is warranted to try to unravel them.

Bone is a metabolically active tissue and responds to its loading environment. Accordingly, exposure to heavy body weight or large increases in weight, especially over a lifetime, may result in adaptations to vertebral, and, hence spinal, shape. Studies have shown positive associations between body mass index (BMI) and lumbar lordosis in young adults aged 18–25 and in post-menopausal women [7–9]. However, not all current evidence is consistent as other studies have found limited or no evidence of an association [10, 11]. A limitation of these previous studies is that BMI was only measured once. Consequently, it is impossible to establish whether prolonged exposure to a high BMI or gains in adiposity during different phases of adulthood could be related to compensatory changes in posture. Clinically, a high BMI has been implicated in low back pain prevalence (LBP) [12], due to both mechanical and metabolic effects. For example, Urquhart and colleagues [13] demonstrated a metabolic effect of high fat mass on increasing intensity of low back pain.

In another study, women were shown to compensate for the destabilising anterior shift in the centre of mass relative to the hips during pregnancy by extending their lower back (thus increasing their lordosis) [14]. Similarly, increased central adiposity (represented by waist circumference (WC)) has been suggested to play a role in differences in spino-pelvic parameters between obese and non-obese individuals [11, 15]. A recent study of 300 Nigerian adults (18–65 years old) found that BMI and waist-hip-ratio (WHR) were related to greater lumbar lordosis, sacral inclination and lumbosacral angle [9]. A radiographic study of 200 Mexican adults found no significant differences in spino-pelvic parameters (including lumbar lordosis and pelvic incidence) between different categories of BMI or with presence of elevated central adiposity [11]. Research into the relationship between sagittal spinal shape and WC is limited and rarely include distinctions between central adiposity and overall overweight. However, additional investigations of WC and other components of body composition may help clarify any relationships between BMI and spine shape; by discriminating between contributions from loading, differences in fat distribution and potential metabolic factors.

Another limitation of previous studies is that they have used external measurements of lumbar lordosis [8–10] or defined lordosis by a single angle between vertebral endplates [7, 11]. Statistical shape modelling (SSM), which uses principal components analysis, is a more comprehensive, precise and useful tool for quantitatively describing spinal morphology from medical images [16, 17]. SSM allows the entire shape of the spine to be modelled rather than comparing a combination of angles, which are often correlated and which may miss important information relating to the entire curvature.

Using data from a large, nationally representative birth cohort, we aimed to investigate the associations of spine shapes, characterised using SSM, at age 60–64 with: 1) contemporaneous measures of BMI, waist circumference and body composition (i.e. lean mass index, fat mass index and android:gynoid fat mass ratio); 2) prior BMI and waist circumference and their changes during different periods of adulthood and; 3) length of exposure to overweight.

## Methods

### Study participants

The Medical Research Council (MRC) National Survey of Health and Development (NSHD) is based on a socially stratified sample of 5362 single legitimate births in March 1946 in Scotland, England and Wales [18, 19]. Between 2006 and 2010 (at 60–64 years), eligible participants known to be alive and living in England, Wales and Scotland were invited for an assessment at one of six clinical research facilities (CRF) or to be visited at home by a research nurse. Of the 2856 invited, 2229 were assessed of whom 1690 attended a CRF. All participants who attended a CRF were invited to undergo a DXA scan of the lumbar spine. Participants were imaged in a supine position in all centres except one, where they were scanned in lateral decubitus due to a fixed C-arm in the scanner. Images were acquired on Hologic QDR 4500 Discovery DXA scanners (Hologic Inc, Bedford, MA) by trained technicians using a standardised protocol and were quality assurance tested [20].

The study was conducted according to the principles expressed in the Declaration of Helsinki. All participants provided written informed consent, and the study received ethical approval from the Central Manchester Research Ethics Committee (07/H1008/245) and the Scottish A Research Ethics Committee (08/MRE00/12).

### Statistical shape modelling

Of the 1690 participants who attended a CRF, 1601 had a lumbar spine DXA scan. Of the 1601 images acquired, 72 were excluded for the following reasons: unable to clearly determine all vertebral outlines (41 removed), scanning artefacts (23 removed), incomplete images (5 removed), presence of metalwork (2 removed) and excessive axial rotation (1 removed). Therefore, a total of 1529 spine images were used in building the statistical shape modelling (SSM). SSM of these images has been described in detail elsewhere [21]. Briefly, an 89-point template was constructed using custom SSM software (Shape, University of Aberdeen) which described spine shape from the tenth thoracic (T10) to fifth lumbar (L5) vertebrae, marking the vertebral body outlines. Point outlines in each image were scaled, rotated and translated (Procrustes transformation) to normalise the scale, thus removing size differences. Finally, principal components analysis identified orthogonal modes of variation in spine shape in which each mode describes variations that occur in combination. Each mode explains a percentage of variation ranked in reverse order (largest first) from Mode 1. Each mode has a mean of zero and a standard deviation of 1 and each image is assigned a score in units of standard deviation.

Statistical and clinical significance of spine shape modes (SMs) was determined by analysing a scree plot showing the percentage of variance explained by each mode individually [21].

### Anthropometric measurements

Height, weight and waist circumference (WC; taken at the midpoint between the costal margin and iliac crest) were measured by nurses using standardised protocols at ages 36, 43, 53 and 60–64 years. BMI was calculated at each age as weight (kg)/height^2^ (m)^2^.

Participants were also categorised into 5 groups representing the age they were first recorded as overweight (i.e. BMI ≥ 25 kg/m^2^). These groups were: overweight at age: 36; 43; 53; 60–64 and never overweight.

Body composition was measured from supine whole body DXA scans at age 60–64. These included measures of total fat and lean mass, abdominal fat mass and hip fat mass, all converted to kilograms. Whole body (excluding head) lean mass index (LMI) was calculated as lean mass (kg)/height^2^ (m)^2^ and fat mass index (FMI), calculated as fat mass (kg)/height^2^ (m)^2^. Android:gynoid fat mass ratio (AGFMR) was derived by dividing abdominal fat mass (kg) by hip fat mass (kg) [22].

In order to facilitate comparisons of effect sizes across ages and measures we standardised all BMI, WC and body composition measures to a mean of 0 and standard deviation (SD) of 1. We did this stratified by sex in order to take account of sex differences in the distributions of our anthropometric measures and hence facilitate comparisons of associations by sex.

### Statistical analysis

Analyses were carried out using linear regression models. All analyses were conducted using STATA v14.1. As sex-differences in spine shape have been reported in a previous analysis [21], all analyses were conducted separately by sex. To formally assess differences in associations between men and women, sex by body size interaction terms were tested in multiple regression models including both men and women.

First, we examined cross-sectional associations of each spine mode (SM 1–4) with each anthropometric measure (i.e. BMI, WC, LMI, FMI, and AGFMR (aim 1)). In these analyses LMI and FMI were mutually adjusted for each other to distinguish the associations with these separate components of body size. There is a correlation between these measures resulting from adaptive physiological responses whereby people with higher fat mass also accrue greater levels of lean mass [23]. The other anthropometric measures were not mutually adjusted for each other in order to avoid multicollinearity (see S1 Table for correlations between measures at age 60–64). We then investigated whether BMI and WC measured at younger ages in adulthood (i.e. ages 36, 43 and 53) were associated with each SM (aim 2). In all of these models deviations from linearity were assessed by including quadratic terms and, where this was evident, tests for linear trend across sex-specific fifths were undertaken.

In order to examine whether there were differential effects of gains in BMI and waist circumference during different age intervals in adulthood (aim 2), the conditional changes in BMI and WC between ages 36 to 43, 43 to 53, and 53 to 60–64 were calculated. This was performed by regressing each BMI or WC measure on the earlier measure(s) for each sex and calculating the residuals. Residuals were standardised (0 mean and 1 SD) to ensure comparability between different age intervals. Linear regression models that included the standardised residuals for all three intervals of change and each SM were run using the sample with complete data on these variables, and differences in coefficients were formally tested using Wald tests.

In a final set of models, we examined whether length of exposure to overweight was associated with each SM, with never overweight as the reference category (aim 3), first in unadjusted models. We then adjusted for current BMI in order to test how much of the association was due to the cross-sectional association with current BMI. Tests for deviation from linearity across the overweight categories were performed using likelihood ratio tests (LRT). For each linear regression model, residuals were inspected to ensure that model assumptions were met.

## Results

**Fig 1:**
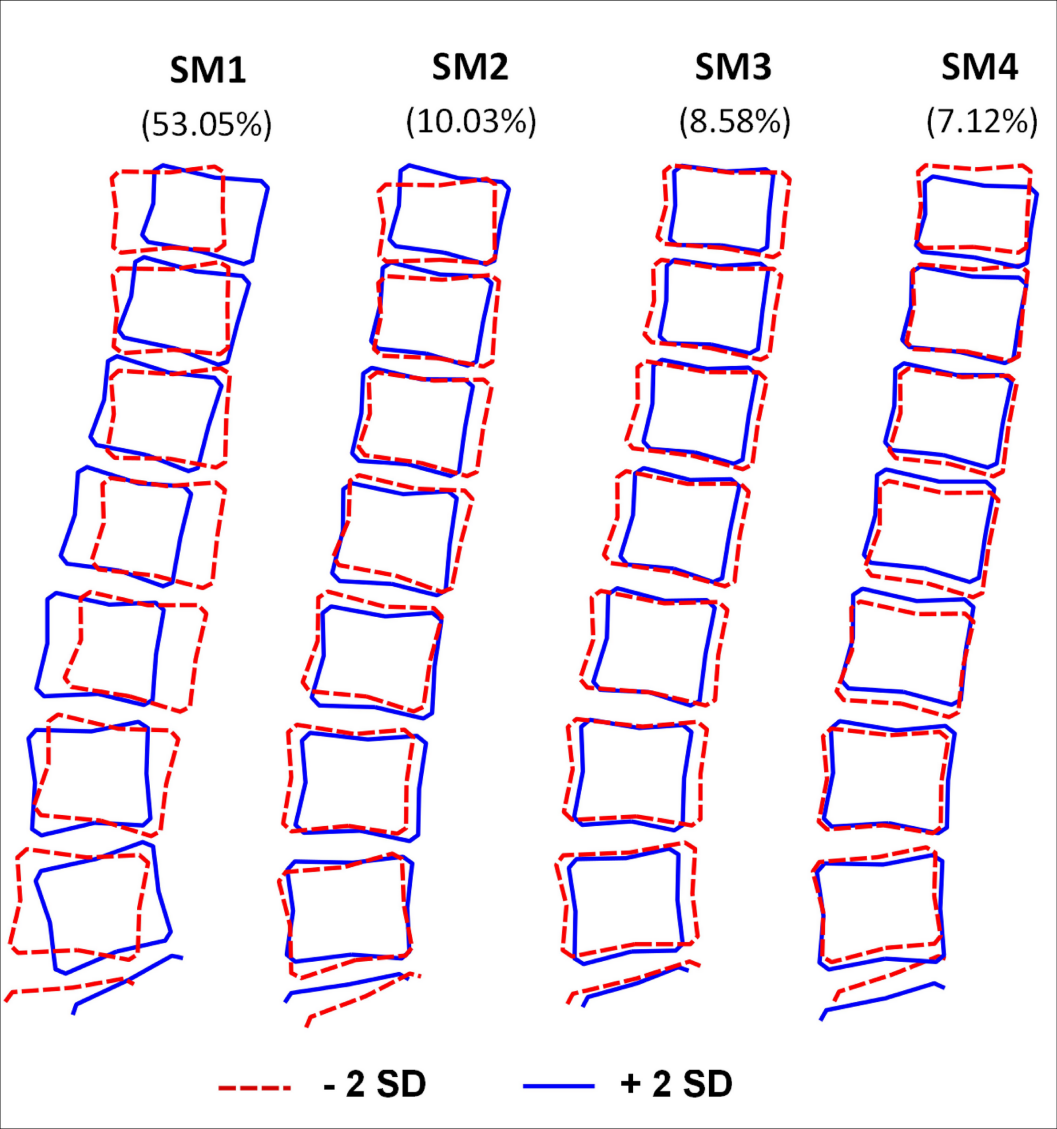
The first four modes (SM1-SM4) produced by statistical shape modelling of the spine. This describes how the shape changes when each mode is varied by two standard deviations in the negative (-2SD, red dashed line) and positive (+2SD, solid blue line) directions. Percentage of variance explained by each mode is shown in brackets.

The characteristics of the study sample are shown in Table 1. Mean BMI and WC increased with age and there were sex differences at all ages. At age 60–64, men had a higher LMI and a higher AGFMR whereas women had a higher mean FMI (Table 1). The first four modes each explained more than 5% of the total variance and together accounted for 79% of the variation within the sample. A detailed description of the first four modes is provided elsewhere [21] but they are visually depicted in Fig 1.

**Table 1:** Characteristics of participants from the MRC National Survey of Health and Development with data on spine shape at age 60–64, stratified by sex.

Variables	Males		Females		*P*-value*
	N		N		
Sex, N (%)	740	(48.4)	789	(51.6)	
Age at nurse visit (y); mean (SD)	740	63.2 (1.17)	789	63.3 (1.09)	.145
*Spine shape modes; mean (SD)*	740		789		
SM1		-0.08 (0.97)		0.07 (1.02)	.004
SM2		0.01 (1.02)		-0.01 (0.99)	.59
SM3		-0.50 (0.97)		0.47 (0.77)	< .001
SM4		0.05 (0.97)		-0.04 (1.03)	.07
*BMI (kg/m*^*2*^*); mean (SD)*					
36y	662	24.4 (2.86)	725	22.9 (3.05)	< .001
43y	693	25.3 (3.05)	746	24.4 (3.67)	< .001
53y	688	27.1 (3.63)	762	26.5 (4.42)	.0124
60-64y	739	27.7 (3.90)	789	27.2 (4.62)	.0125
*Waist circumference (cm); mean (SD)*					
36y	664	88.2 (8.33)	726	75.1 (9.59)	< .001
43y	689	90.4 (8.79)	750	75.9 (8.77)	< .001
53y	689	96.6 (9.68)	765	83.5 (10.9)	< .001
60-64y	738	100.2 (10.4)	787	90.7 (11.5)	< .001
*DXA measurements at 60-64y; mean (SD)*					
Fat mass index (kg/m^2^)	700	7.73 (2.27)	744	10.8 (3.20)	< .001
Lean mass index (kg/m^2^)	700	17.5 (1.97)	744	14.1 (1.76)	< .001
Android: gynoid fat mass ratio	732	0.65 (0.15)	781	0.44 (0.12)	< .001
*Age first overweight(y); n (%)*	642		691		< .001
Never overweight		122 (19.0)		191 (27.6)	
Overweight 36y		259 (40.3)		141 (20.4)	
Overweight 43y		109 (15.0)		120 (17.4)	
Overweight 53y		105 (16.4)		169 (24.5)	
Overweight 60-64y		47 (7.3)		70 (10.1)	

BMI: Body Mass Index; SM: spine mode; DXA: Dual Energy X-Ray Absorptiometry

*comparison of sexes using student *t*-test or chi-square tests as appropriate

### Spine mode 1

SM1 described differences in the total amount of curvature within the spine (L5 to T10), with positive (higher) scores representing a more lordotic curvature and negative (lower) scores representing a flatter or less lordotic lumbar curve with slight thoracic kyphosis. In men (*P*<0.05 for sex interactions), a greater LMI was associated with a greater lordosis (higher SM1 scores) whereas greater FMI and AGFMR were associated with a straighter spine (lower SM1 scores) (Table 2). No associations were observed between BMI or WC at any age and SM1 (Table 2, S2 Table). However, greater gains in BMI and WC between ages 36 and 43 were weakly associated with lower SM1 scores in men, (Table 3). There were no associations between age first overweight and SM1 scores in either sex (Table 4).

**Table 2:** Cross-sectional associations between BMI, WC and body composition and spine modes at age 60–64.

	Men, n = 698		Women, n = 742	
Per 1SD increase	β (95%CI)	*P*-value	β (95%CI)	*P*-value
**SM1**				
BMI	-0.004 (-0.08, 0.073)	.9	-0.007 (-0.096, 0.082)	.9
WC	0.0003 (-0.076, 0.077)	.99	0.043 (-0.042, 0.128)	.3
LMI ¶*	0.198 (0.11, 0.286)	< .001	-0.019 (-0.12, 0.082)	.7
FMI ¶*	-0.18 (-0.27, -0.091)	< .001	0.015 (-0.091, 0.121)	.8
AGFMR*	-0.144 (-0.218, -0.071)	< .001	-0.014 (-0.09, 0.061)	.7
**SM2**				
BMI	-0.084 (-0.162, -0.005)	.04	-0.029 (-0.112, 0.055)	.5
WC	-0.069 (-0.147, 0.009)	.08	0.013 (-0.067, 0.093)	.7
LMI ¶	-0.041 (-0.132, 0.05)	.4	-0.001 (-0.096, 0.094)	.99
FMI ¶	-0.055 (-0.148, 0.037)	.2	-0.023 (-0.123, 0.076)	.6
AGFMR	-0.053 (-0.129, 0.022)	.2	0.028 (-0.043, 0.099)	.4
**SM3**				
BMI	-0.126 (-0.202, -0.051)	.001	-0.114 (-0.18, -0.049)	.001
WC	-0.125 (-0.2, -0.049)	.001	-0.117 (-0.18, -0.055)	< .001
LMI ¶	-0.264 (-0.35, -0.177)	< .001	-0.223 (-0.296, -0.149)	< .001
FMI ¶	0.111 (0.023, 0.199)‡	.013	0.093 (0.016, 0.169)	.02
AGFMR	0.018 (-0.056, 0.092)	.6	-0.02 (-0.076, 0.037)	.5
**SM4**				
BMI	0.011 (-0.065, 0.087)	.8	0.085 (-0.003, 0.173)	.06
WC	0.02 (-0.056, 0.096)	.6	0.12 (0.036, 0.204)	.005
LMI ¶	0.005 (-0.083, 0.094)	.9	0.089 (-0.011, 0.19)	.08
FMI ¶	0.010 (-0.080, 0.100)	.8	0.007 (-0.098, 0.112)	.9
AGFMR	-0.007 (-0.081, 0.066)	.8	0.085 (0.01, 0.16)	.03

(Analyses are restricted to those with data on BMI, WC, LMI, FMI, AGFMR and spine mode scores at age 60–64 (n = 1,440)

BMI: body mass index; WC: waist circumference; LMI: lean mass index; FMI: fat mass index; AGFMR; android (abdominal): gynoid (hip) fat mass ratio

¶ adjusted for each other

* sex interactions, *P* ≤.05

‡ non-linear relationship (LRT for quadratic term, *P* = 0.002) and overall LRT for trend across sex-specific quintiles for FMI (*P* = 0.0189)

**Table 3:** Associations of gains in BMI and waist circumference during different age intervals with spine modes at age 60–64.

	Men		Women	
Interval of BMI/ WC change (age (y))	β (95%CI)	*P*-value†	β (95%CI)	*P*-value†
**BMI**	n = 606		n = 682	
SM1				
36–43*	-0.083 (-0.166, -0.001)	.05	0.037 (-0.047, 0.121)	.4
43–53	-0.038 (-0.119, 0.042)	.3	-0.028 (-0.106, 0.05)	.5
53–60–64	0.047 (-0.036, 0.131)^a^	.3	-0.003 (-0.084, 0.078)	.9
SM2				
36–43	-0.043 (-0.126, 0.039)	.3	-0.03 (-0.112, 0.053)	.5
43–53	0.042 (-0.038, 0.122)	.3	0.051 (-0.026, 0.127)	.2
53–60–64	0.041 (-0.042, 0.123)	.3	-0.021 (-0.1, 0.058)	.6
SM3				
36–43	-0.009 (-0.09, 0.073)	.8	0.023 (-0.042, 0.087)	.5
43–53*	0.092 (0.013, 0.171)	.02	-0.003 (-0.062, 0.056)	.9
53–60–64	0.008 (-0.074, 0.09)	.8	0.021 (-0.041, 0.082)	.5
SM4				
36–43	-0.018 (-0.1, 0.065)	.7	-0.006 (-0.092, 0.079)	.9
43–53	-0.037 (-0.117, 0.043)	.4	-0.03 (-0.109, 0.049)	.5
53–60–64	-0.024 (-0.107, 0.059)	.6	-0.017 (-0.099, 0.065)	.7
**WC**	n = 607		n = 689	
SM1				
36–43*	-0.073 (-0.15, 0.005)	.07	0.066 (-0.014, 0.147)	.1
43–53	-0.054 (-0.133, 0.025)	.2	-0.004 (-0.078, 0.071)	.9
53–60–64	0.052 (-0.028, 0.132)^b^	.2	0.051 (-0.027, 0.13)	.2
SM2				
36–43	-0.042 (-0.12, 0.036)	.3	0.03 (-0.049, 0.109)	.5
43–53	0.016 (-0.064, 0.096)	.7	0.006 (-0.067, 0.079)	.9
53–60–64	0.060 (-0.021, 0.141)	.1	-0.003 (-0.08, 0.074)	.9
SM3				
36–43*	0.039 (-0.038, 0.115)	.3	-0.087 (-0.148, -0.026)	.005
43–53	0.015 (-0.064, 0.093)	.7	-0.023 (-0.079, 0.034)	.4
53–60–64	-0.013 (-0.092, 0.066)	.7	-0.023 (-0.083, 0.036)	.4
SM4				
36–43	0.056 (-0.022, 0.134)	.2	0.058 (-0.023, 0.14)	.2
43–53	-0.011 (-0.091, 0.069)	.8	0.021 (-0.055, 0.096)	.6
53–60–64	-0.014 (-0.095, 0.066)	.7	0.013 (-0.067, 0.092)	.7

BMI, body mass index; WC, waist circumference

†Wald’s *P-*value

* sex interactions, p≤.05

^a^Association between BMI gain from 53 to 60-64y and SM1 was larger than the association from 36 – 43y, (Wald test *P-*value of the difference between the two coefficients = 0.03)

^b^Association between waist circumference gain from 53 to 60-64y and SM1 was larger than the association from 36 – 43y, (Wald test *P-*value of the difference between the two coefficients = 0.03)

**Table 4:** Associations of age at first overweight with spine modes at age 60–64.

		SM1	SM2	SM3	SM4
		β (95%CI)	β (95%CI)	β (95%CI)	β (95%CI)
***Unadjusted†***					
**Men** (n = 642)					
Overweight 36y		0.027 (-0.182, 0.235)	-0.315 (-0.529, -0.101)	-0.277 (-0.484, -0.071)	0.011 (-0.199, 0.220)
Overweight 43y		0.030 (-0.220, 0.280)	-0.287 (-0.544, -0.03)	-0.029 (-0.277, 0.219)	-0.078 (-0.329, 0.174)
Overweight 53y		-0.088 (-0.341, 0.164)	0.067 (-0.193, 0.326)	-0.114 (-0.365, 0.137)	-0.208 (-0.462, 0.046)
Overweight 60-64y		0.234 (-0.092, 0.559)	-0.02 (-0.354, 0.315)	0.048 (-0.275, 0.372)	0.126 (-0.202, 0.453)
*P*-value trend (except **)		.7	.002**	.024**	.4
**Women** (n = 691)					
Overweight 36y		-0.038 (-0.26, 0.185)	-0.057 (-0.273, 0.159)	-0.262 (-0.428, -0.096)	0.161 (-0.063, 0.385)
Overweight 43y		-0.124 (-0.357, 0.109)	-0.153 (-0.38, 0.074)	-0.226 (-0.401, -0.051)	0.123 (-0.112, 0.359)
Overweight 53y		0.020 (-0.192, 0.231)	0.011 (-0.194, 0.217)	-0.064 (-0.222, 0.094)	0.014 (-0.199, 0.228)
Overweight 60-64y		-0.01 (-0.289, 0.27)	-0.008 (-0.28, 0.264)	0.059 (-0.151, 0.268)	0.008 (-0.274, 0.291)
*P*-value trend (except **)		.96	.98	.003**	.9
*P*-for sex interaction		.5	.3	.6	.4
***Adjusted for current BMI†***					
**Men** (n = 642)					
Overweight 36y		0.116 (-0.178, 0.409)	-0.299 (-0.600, 0.003)	-0.184 (-0.475, 0.107)	-0.051 (-0.346, 0.244)
Overweight 43 y		0.089 (-0.196, 0.374)	-0.276 (-0.569, 0.016)	0.033 (-0.250, 0.315)	-0.118 (-0.405, 0.168)
Overweight 53y		-0.042 (-0.317, 0.233)	0.075 (-0.207, 0.357)	-0.065 (-0.338, 0.208)	-0.240 (-0.517, 0.036)
Overweight 60-64y		0.270 (-0.067, 0.608)	-0.013 (-0.360, 0.333)	0.087 (-0.247, 0.422)	0.100 (-0.239, 0.439)
*P*-value trend (except **)		.7	.024**	.3	.4
**Women** (n = 691)					
Overweight 36y		-0.080 (-0.406, 0.247)	0.036 (-0.282, 0.353)	-0.229 (-0.473, 0.016)	0.139 (-0.190, 0.469)
Overweight 43 y		-0.154 (-0.443, 0.135)	-0.087 (-0.368, 0.194)	-0.202 (-0.419, 0.014)	0.107 (-0.184, 0.399)
Overweight 53y		-0.003 (-0.249, 0.243)	0.06 (-0.179, 0.299)	-0.046 (-0.231, 0.138)	0.003 (-0.246, 0.252)
Overweight 60-64y		-0.027 (-0.324, 0.270)	0.03 (-0.258, 0.319)	0.073 (-0.150, 0.295)	-0.001 (-0.301, 0.299)
*P*-value trend (except **)		.9	.7	.08**	.6
*P*-for sex interaction		.5	.3	.6	.5

†reference group: never overweight (BMI <25kg/m^2^)

**P-value for test of heterogeneity across groups when there was evidence of a deviation from a linear trend

### Spine mode 2

Having defined the overall curvature by SM1, SM2 described differences in the distribution of curvature along the length of the spine. Positive (higher) scores represented an evenly distributed curvature whereas negative (lower) scores represented a more uneven distribution, more S-shaped or snaking. In men, higher current BMI was associated with lower SM2 scores but this was not reflected in LMI, FMI or AGFMR. No cross-sectional associations were observed in women (Table 2). When BMI and WC earlier in adulthood were examined, there was evidence of associations between higher BMI at younger ages and lower SM2 scores in both sexes and between higher WC and lower SM2 scores in men, with stronger effects at ages 36 and 43 (S2 Table). There were no associations between gains in BMI or WC and SM2 in either sex (Table 3). There was evidence that the associations between age at first overweight and SM2 in men was non-linear. Consistent with the finding of stronger associations between BMI at younger ages and SM2, there was evidence that men with onset of overweight at 36 and 43 had lower SM2 scores compared with those never overweight and this was only partially attenuated after adjustment for current BMI (Table 4). There was no evidence of any sex interactions.

### Spine mode 3

SM3 described variations in antero-posterior vertebral diameter relative to vertebral height (a-p aspect ratio). Negative (lower) scores represented larger relative diameters and positive (higher) scores smaller relative diameters. In both men and women, higher current BMI, WC and LMI were all associated with larger a-p aspect ratios (lower SM3 scores) whereas higher FMI was associated with higher SM3 scores (Table 2). Higher BMI and WC at younger ages were more strongly associated with lower SM3 scores (S2 Table). Although there was some evidence of associations of BMI gain between ages 43 and 53 with SM3 in men (*P*<0.05 sex interaction) and of WC gain between ages 36 and 43 with SM3 in women (*P*<0.05 sex interaction), this was weak (Table 3). The relationship between age at overweight and SM3 scores was not linear. Onset of overweight at 36 in men and at 36 and 43 in women was associated with lower SM3 scores, with some attenuation of the differences after adjusting for current BMI, particularly among men (Table 4).

### Spine mode 4

SM4 captured subtle variations in the evenness of disc and vertebral thicknesses. Positive (higher) scores represented thicker and relatively uniform disc heights with slightly larger posterior vertebral heights, especially in the lower lumbar region. In comparison, negative (lower) scores indicated disc narrowing, especially posteriorly with a compensatory anterior vertebral narrowing, again slightly clearer in the lower lumbar region. Higher current WC and AGFMR were weakly associated with higher SM4 scores in women but no associations were observed with BMI, LMI or FMI (Table 2). Among women, higher BMI and WC at all younger ages were associated with higher SM4 scores (S2 Table). No associations were observed between BMI or WC gain or age first overweight and SM4 in either sex (Tables 3 and 4). There was no evidence of any sex interactions.

## Discussion

We have explored the relationships between spinal shape in early old-age (60–64 years old), characterised by SSM, and changes in BMI and WC (as markers of total and central adiposity, respectively) throughout adult life in the MRC National Survey of Health and Development. The majority of variation in spinal shape was captured in four modes, which described morphological variations similar to those in previous SSM studies of the spine [1, 2]. Total and central adiposity in early adulthood appeared to have the strongest relationships with current spine shape. Shape variation associated with greater BMI and WC was found mainly in the evenness of the lumbar curvature and in the anterior-posterior vertebral diameter relative to the vertebral height rather than in the gross curvature of the spine.

Although current BMI and WC were associated with uneven (or snaking) spinal curvatures (SM2) and larger vertebral a-p aspect ratios (SM3) associations were stronger with measures in earlier adulthood. Thus, having a greater BMI, a larger waist circumference and becoming overweight earlier in adulthood appeared to be more strongly related to an increasing unevenness of the lumbar lordosis and the size of the vertebrae. There were no associations between LMI, FMI or AGFMR and SM2 which we had hoped may clarify whether this association could be explained by a metabolic effect of adipose tissue or greater loading from increased weight. The snakier curvatures (SM2) seen in men with larger WC could be a consequence of the weight of the abdomen bringing the lower spine forward and causing the lumbar lordosis to be concentrated in the lower lumbar spine. However, we are unable to discriminate between anterior and posterior contributions of WC. It is acknowledged that as WC is strongly positively correlated with BMI it also acts as a marker of overall body size. In situations where WC is acting in this way we would expect it to mirror results for BMI. Here it does this only partially and is, therefore, suggestive of something specific to loading due to weight around the abdomen. Previous studies have suggested a more lordotic posture in pregnant women and overweight adults but comparison with these studies is difficult; they all used external measures of lordosis that are incapable of capturing the evenness of curvature whereas here we measure the vertebral body positions and obtain more sophisticated measures of overall curvature and the distribution of curvature.

The consistent relationship between high BMI and large vertebral a-p aspect ratios (SM3) appears to support our earlier predictions that an osteogenic response to weight may be involved. Although measures of diameter are relative to height this still indicates a broadening of the vertebrae which may be an attempt to maintain a constant stress (load divided by area) in the face of increasing loads. It is interesting to note that lean mass and fat mass had contrasting associations with vertebral body aspect ratios and women with larger increases in WC earlier in adulthood (ages 36–43) had smaller vertebral a-p diameters relative to height. Whereas fat mass may have both endocrine and weight-bearing roles, lean mass is assumed to be more important for forces applied to the skeleton. Individuals who have greater lean mass tend to be more physically active, than those with greater fat mass, therefore exercise and muscle tension may have subjected the vertebrae to more loading over time and elicited an osteogenic effect on vertebral bone [24, 25]. Future analysis of osteoporosis (OP) prevalence in this group will help to identify whether or not having a larger a-p aspect ratio is beneficial for reducing OP risk.

The overall curviness of the spine (SM1) in this cohort was not related to either current overall or central measures of adiposity and was not associated with how long individuals had been overweight during adulthood. These data extend a small study (n = 36) of external spine curvatures that found no difference in lumbar lordosis between obese and normal weight participants but found obese participants to have a 16° greater thoracic kyphosis [26]. Our results suggest that men who had greater lean mass were more likely to have a curvier lumbar spine while those with more fat mass and central adiposity had a flatter lordosis. Postural changes may explain the flatter lumbar curve in men with central adiposity. The clinical definition of a sway-back posture, where the trunk leans backward relative to the pelvis, includes a reduced lumbar lordosis and posterior pelvic tilt [3, 27, 28] and has been related to increased fat infiltration in the lumbar erector spinae and multifidus muscles [29]. It is possible that these men with central adiposity adopt a sway-back posture in standing which has manifested in the recumbent DXA scans by affecting the intrinsic spinal shape evident in all postures [1].

Spinal shapes presenting with thinner and more wedged lower lumbar disc spaces (SM4), were associated with greater overall and central adiposity (BMI and WC) at younger ages, especially in women. Although the amount of variation described by Mode 4 is small, the relationship with central adiposity, as indicated by WC and AGFMR, remained into early old age (60–64 years) in women and the clinical significance of this variation is not known. Disc thinning is associated with degeneration and with osteoarthritis. Although study of these disorders is beyond the scope of these analyses, future studies will explore whether these variations in spine shape are associated with pathological changes.

A strength of this study is that the data come from a large, prospective population-based study. There are few opportunities to explore life course factors in musculoskeletal physiology and pathology and this group presents a unique opportunity to explore the role of body weight and, more importantly, the distribution of fat that is increasingly recognised as being a risk factor for many disorders. The long-term nature of this study enables repeated measures of adiposity (BMI and WC) and direct body composition and begins to separate the effects of chronic from late-onset obesity.

There are a number of limitations to this study of the spine. The first of these is that one of the six imaging centres had a fixed C-arm in the scanner. This did not allow lateral spine imaging to be acquired in a supine position and therefore participants were lying on their side, which had the potential to affect spine positioning. However, when we adjusted for imaging centre in our analytical models we found no effect on the results [21].

Secondly, DXA imaging is two dimensional in nature and was chosen as the best available method at the time of scanning as it provides both images and measures of bone density with a low radiation dose. Indeed, it is still a preferred method for large studies of this kind. There is, however, potential for rotation errors. We minimised this risk by excluding images with extreme rotation, through visual inspection of images. Images where posterior vertebral elements were seen to overlap on the vertebral body were excluded.

Thirdly, in standing, geometrical measures of lumbar lordosis have been related to pelvic incidence (which is independent of pelvic tilt) and sacral slope [30, 31]; a larger lordosis being related to a greater pelvic incidence and sacral slope. However, this is not something we can measure from the field of view in the lateral spine DXA scans available to us. It is unclear whether this relationship would remain in the supine postures which were adopted here or whether it would it affect the overall shape as measured by SSM. When pelvic tilt was measured externally, Delisle and colleagues [32] found no effect on the lumbar angles when participants tilted their pelvis backwards, which is what would be expected to happen when lying supine [33].

In summary, total and central adiposity were associated with aspects of thoracolumbar spinal shape in men and women aged 60–64. Being overweight and having greater central adiposity earlier in life were particularly important and point to an effect of increased mechanical loading on the spine over time. A potential metabolic effect of greater adiposity over time is also possible and requires further investigation.

## Supporting information

S1 Table Correlations between body composition measures at age 60–64. (PDF)

S2 Table Associations of BMI and waist circumference across adulthood with spine modes. (PDF)
