# Supplementary material for: Body mass index and waist circumference in early adulthood are associated with thoracolumbar spine shape at age 60-64: The Medical Research Council National Survey of Health and Development
Source: PLoS One. 2018 Jun 14;13(6):e0197570. doi: 10.1371/journal.pone.0197570 (PMC6002244; doi:10.1371/journal.pone.0197570)
Supplement: S1 Table — (PDF) [file pone.0197570.s001.pdf]

Body mass index and waist circumference in early adulthood are associated with thoracolumbar spine shape at age 60-64: the Medical Research Council National Survey of Health and Development

Anastasia V. Pavlova, Stella G. Muthuri, Rachel Cooper, Fiona R. Saunders, Jennifer S. Gregory, Rebecca J. Barr, Kathryn R. Martin, Judith E. Adams, Diana Kuh, Rebecca J. Hardy, Richard M. Aspden

Supplementary information

Table S1: Correlations between body composition measures at age 60-64.

	BMI	WC	LMI	FMI	AGFMR
**BMI**	1	0.86	0.83	0.95	0.56
**WC**	0.88	1	0.72	0.81	0.66
**LMI**	0.86	0.71	1	0.64	0.43
**FMI**	0.90	0.85	0.57	1	0.54
**AGFMR**	0.52	0.54	0.37	0.53	1

Correlation coefficients are presented in white for men and in grey for women. BMI: body mass index; WC: waist circumference; LMI: lean mass index; FMI: fat mass index; AGFMR; android (abdominal): gynoid (hip) fat mass ratio.
