# Supplementary material for: Body mass index and waist circumference in early adulthood are associated with thoracolumbar spine shape at age 60-64: The Medical Research Council National Survey of Health and Development
Source: PLoS One. 2018 Jun 14;13(6):e0197570. doi: 10.1371/journal.pone.0197570 (PMC6002244; doi:10.1371/journal.pone.0197570)
Supplement: S2 Table — (PDF) [file pone.0197570.s002.pdf]

Body mass index and waist circumference in early adulthood are associated with thoracolumbar spine shape at age 60-64: the Medical Research Council National Survey of Health and Development

Anastasia V. Pavlova, Stella G. Muthuri, Rachel Cooper, Fiona R. Saunders, Jennifer S. Gregory, Rebecca J. Barr, Kathryn R. Martin, Judith E. Adams, Diana Kuh, Rebecca J. Hardy, Richard M. Aspden

Supplementary information

Table S2: Associations of BMI and waist circumference across adulthood with spine modes.

Per 1SD increase	Men<br>β (95%CI)	P value†	Women<br>β (95%CI)	P value†
**BMI**				
**SM1**				
36y	0.005 (-0.069 , 0.078)	.9	-0.005 (-0.08 , 0.069)	.9
43y	-0.026 (-0.098 , 0.046)	.5	0.01 (-0.063 , 0.083)	.8
53y	-0.034 (-0.107 , 0.039)	.357	0.015 (-0.058 , 0.087)	.7
60-64y	-0.001 (-0.071 , 0.069)	.973	-0.01 (-0.082 , 0.061)	.8
**SM2**				
36y	-0.147 (-0.222 , -0.071)	<.001	-0.066 (-0.137 , 0.005)	.07
43y	-0.114 (-0.188 , -0.04)	.003	-0.081 (-0.153 , -0.01)	.025
53y	-0.085 (-0.16 , -0.01)	.026	-0.038 (-0.109 , 0.033)	.3
60-64y	-0.074 (-0.148 , -0.001)	.047	-0.035 (-0.104 , 0.034)	.3
**SM3**				
36y	-0.168 (-0.241 , -0.096)	<.001	-0.137 (-0.192 , -0.082)‡	<.001
43y	-0.184 (-0.256 , -0.113)	<.001	-0.116 (-0.171 , -0.061)‡	<.001
53y	-0.099 (-0.171 , -0.027)	.007	-0.107 (-0.162 , -0.052)	<.001
60-64y	-0.119 (-0.189 , -0.049)	.001	-0.091 (-0.145 , -0.037)	.001
**SM4**				
36y	0.055 (-0.019 , 0.129)	.1	0.101 (0.026 , 0.175)	.008
43y	0.019 (-0.054 , 0.092)	.6	0.088 (0.014 , 0.162)	.02
53y	0.006 (-0.066 , 0.079)	.9	0.069 (-0.004 , 0.143)	.07
60-64y	-0.006 (-0.076 , 0.065)	.9	0.067 (-0.004 , 0.139)	.07
**WC**				
**SM1**				
36y	0.023 (-0.051 , 0.096)	.5	-0.046 (-0.12 , 0.029)‡	.2
43y	-0.036 (-0.109 , 0.036)	.3	0.028 (-0.044 , 0.101)	.4
53y	-0.033 (-0.106 , 0.04)	.4	0.008 (-0.065 , 0.08)	.8
60-64y	0.006 (-0.064 , 0.076)	.9	0.021 (-0.051 , 0.093)	.6
**SM2**				
36y	-0.153 (-0.229 , -0.078)	<.001	-0.053 (-0.124 , 0.019)	.2
43y	-0.106 (-0.18 , -0.032)	.005	-0.006 (-0.077 , 0.065)	.9
53y	-0.076 (-0.153 , -0.00003)	.05	0.001 (-0.069 , 0.072)	.97
60-64y	-0.067 (-0.14 , 0.006)	.07	0.022 (-0.047 , 0.09)	.5
**SM3**				
36y*	-0.168 (-0.24 , -0.096)	<.001	-0.051 (-0.107 , 0.005)‡	.07
43y	-0.117 (-0.19 , -0.045)‡	.001	-0.11 (-0.165 , -0.055)	<.001
53y	-0.108 (-0.18 , -0.036)	.003	-0.095 (-0.15 , -0.04)	.001
60-64y	-0.12 (-0.19 , -0.05)	.001	-0.1 (-0.154 , -0.047)	<.001
**SM4**				
36y	0.039 (-0.034 , 0.113)	.3	0.09 (0.016 , 0.164)	.017
43y	0.041 (-0.032 , 0.114)	.3	0.097 (0.023 , 0.171)	.01
53y	0.02 (-0.053 , 0.092)	.6	0.096 (0.023 , 0.168)	.01
60-64y	0.008 (-0.063 , 0.078)	.8	0.101 (0.029 , 0.173)	.006

BMI, body mass index; WC, waist circumference. Models are run on the maximum available N at each age (see table 1). †Wald's p value; *sex-interaction p<.05. ‡ significant quadratic term but deviation from linearity was not confirmed when tests for trend across sex-specific quintiles were undertaken.
